# Inhibition of COP9-signalosome (CSN) deneddylating activity and tumor growth of diffuse large B-cell lymphomas by doxycycline

**DOI:** 10.18632/oncotarget.4193

**Published:** 2015-06-04

**Authors:** Mary Pulvino, Luojing Chen, David Oleksyn, Jing Li, George Compitello, Randy Rossi, Stephen Spence, Vijaya Balakrishnan, Craig Jordan, Brian Poligone, Carla Casulo, Richard Burack, Joel L. Shapiro, Steven Bernstein, Jonathan W. Friedberg, Raymond J. Deshaies, Hartmut Land, Jiyong Zhao

**Affiliations:** ^1^ Department of Biomedical Genetics, University of Rochester Medical Center, Rochester, NY, USA; ^2^ Division of Allergy/Immunology and Rheumatology, University of Rochester Medical Center, Rochester, NY, USA; ^3^ Division of Biology and Biological Engineering, California Institute of Technology, Pasadena, CA, USA; ^4^ Wilmot Cancer Institute, University of Rochester Medical Center, Rochester, NY, USA; ^5^ Department of Pathology, University of Rochester Medical Center, Rochester, NY, USA; ^6^ Division of Hematology, University of Colorado Denver, Aurora, CO, USA; ^7^ Department of Dermatology, University of Rochester Medical Center, Rochester, NY, USA; ^8^ Department of Pathology, Rochester General Hospital, Rochester, NY, USA; ^9^ Howard Hughes Medical Institute, California Institute of Technology, Pasadena, CA, USA

**Keywords:** DLBCL, doxycycline, therapeutic agent, COP-9 signalosome, CSN5

## Abstract

In searching for small-molecule compounds that inhibit proliferation and survival of diffuse large B-cell lymphoma (DLBCL) cells and may, therefore, be exploited as potential therapeutic agents for this disease, we identified the commonly used and well-tolerated antibiotic doxycycline as a strong candidate. Here, we demonstrate that doxycycline inhibits the growth of DLBCL cells both *in vitro* and in mouse xenograft models. In addition, we show that doxycycline accumulates in DLBCL cells to high concentrations and affects multiple signaling pathways that are crucial for lymphomagenesis. Our data reveal the deneddylating activity of COP-9 signalosome (CSN) as a novel target of doxycycline and suggest that doxycycline may exert its effects in DLBCL cells in part through a CSN5-HSP90 pathway. Consistently, knockdown of CSN5 exhibited similar effects as doxycycline treatment on DLBCL cell survival and HSP90 chaperone function. In addition to DLBCL cells, doxycycline inhibited growth of several other types of non-Hodgkin lymphoma cells *in vitro*. Together, our results suggest that doxycycline may represent a promising therapeutic agent for DLBCL and other non-Hodgkin lymphomas subtypes.

## INTRODUCTION

Diffuse large B-cell lymphoma (DLBCL) is the most common type of non-Hodgkin lymphoma (NHL), accounting for about one third of all the cases. Although advances in treatment have greatly improved the outcome of DLBCL patients, approximately 40% of the patients either are refractory to or relapse from the current standard immunochemotherapy R-CHOP (rituximab plus cyclophosphamide, doxorubicin, vincristine, and prednisone) and most of them will die of the disease within two years of diagnosis [1–4]. Therefore, new therapeutic strategies are urgently needed to combat this malignancy. DLBCL comprises a heterogeneous mixture of distinct lymphomas with different clinical outcomes. Gene expression profiling studies have classified DLBCLs into at least three major subgroups: the germinal center B-cell (GCB), the activated B-cell (ABC), and the mediastinal large B-cell (PMBL) DLBCL [5–7]. Virtually all ABC DLBCL, the least curable DLBCL subtype, and a significant fraction of GCB DLBCL exhibit constitutive NF-κB pathway activity [5, 8–12]. Notably, ABC DLBCL cells depend on constitutive NF-κB signaling for proliferation and survival [11, 13–15]. Targeting pathways required for NF-κB activation thus has been proposed as a novel treatment strategy for DLBCL [16, 17].

Doxycycline is an inexpensive, commonly used and well-tolerated antimicrobial agent. In addition to its antibiotic effect, doxycycline possesses various non-antimicrobial activities. These include its well-studied ability to inhibit the activities of various matrix metalloproteinases (MMPs) as well as its inhibition of MMP gene expression. Additionally, doxycycline has been reported to have anti-inflammatory activity as well as potential antineoplastic activity [18–26]. The molecular mechanisms underlying the non-antibiotic activities of doxycycline have remained poorly understood.

The Connectivity Map, which was generated from a collection of genome-wide gene expression profiles of cultured human cells treated with various bioactive small molecules, including FDA-approved drugs, allows the discovery of potential connections between drugs and signaling pathways [27]. In order to identify drugs that inhibit NF-κB target gene expression and may thereby inhibit the proliferation and survival of DLBCL cells, we carried out a Connectivity Map analysis and identified doxycycline as a strong candidate. Here, we demonstrate that doxycycline inhibits proliferation and survival of DLBCL cells *in vitro* as well as tumor growth of DLBCL cells xenografted in mice at concentrations that may be achievable in human sera with a therapeutic dose of the drug, identifying doxycycline as a potential low-cost and safe therapeutic agent for DLBCL and possibly other NHLs. Additionally, our work uncovers CSN5 as a novel target of doxycycline and as a potential target in DLBCL therapy.

## RESULTS

### Connectivity map analysis uncovers doxycycline as an inhibitor of NF-κB signaling

To identify potential inhibitors of NF-κB signaling that may be exploited as therapeutic agents for DLBCL treatment, we queried the Connectivity Map with a set of known NF-κB targets. Notably, among the top hit compounds that potentially inhibit NF-κB signaling from this analysis are members of the tetracycline family of antibiotics, including doxycycline (Table 1).

**Table 1:** Connectivity map database analysis identifies tetracycline family antibiotics as potential NF-κB signaling inhibitors

Rank	cMAP name	Dose	Cell	Score
6100	lymecycline	7 μM	HL60	−1
5636	metacycline	8 μM	HL60	−0.617
5579	rolitetracycline	8 μM	HL60	−0.603
5138	doxycycline	8 μM	HL60	−0.519
5128	tetracycline	8 μM	HL60	−0.518
4768	demeclocycline	8 μM	HL60	−0.452

A high negative connectivity score indicates that the corresponding compound reversed the expression of the query signature. The bottom ranked instance (i.e. “score” = −1, “Rank” = 6100) is the most negatively connected with the query signature. Details about the “Rank” and “Score” can be found at www.broadinstitute.org/cmap/#.

**Figure 1:**
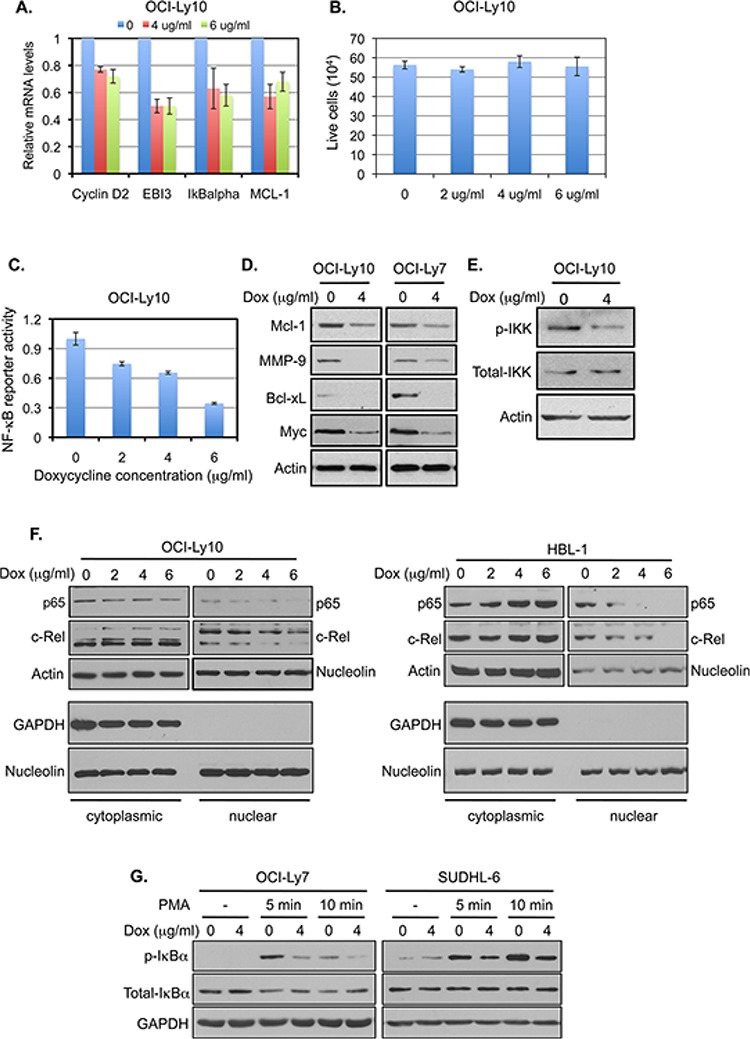
Doxycycline inhibits NF-κB signaling in DLBCL cells **A.** The mRNA levels of the indicated NF-κB targets in OCI-Ly10 cells treated with doxycycline for 12 hrs were analyzed by quantitative RT-PCR. The mean and standard deviation (SD) from triplicate samples are depicted. **B.** The viability of OCI-Ly10 cells, treated as described in (A), was assessed by Trypan blue assay. The assays were carried out in triplicates. **C.** The OCI-Ly10 cells, which carry a stably integrated NF-κB reporter, were treated with indicated concentrations of doxycycline for 22 hrs. The luciferase activity was measured and normalized to protein concentration. **D.** DLBCL cells were treated with the indicated concentrations of doxycycline for 24 hrs. The protein levels of the NF-κB-regulated targets were analyzed by western blotting. Analysis of actin was included as a loading control. **E.** OCI-Ly10 cells were treated with doxycycline for 8 hrs. The levels of the indicated proteins were analyzed by western blotting. **F.** OCI-Ly10 and HBL-1 cells, both ABC-DLBCL cell lines, were treated with doxycycline for 24 hrs. The levels of indicated NF-κB subunits in cytoplasmic and nuclear fractions, prepared as previously described [86], were analyzed by western blotting (top panels). Lack of GAPDH signal in the nuclear fraction (low panels) indicates the clean preparation of the nuclear fraction. Nucleolin, which is present in both cytosolic and nuclear fractions in cancer cells [87], was used as a loading control. **G.** Two GCB-DLBCL cell lines, OCI-Ly7 and SUDHL-6, were treated with the indicated concentrations of doxycycline overnight and stimulated with PMA. The levels of the indicated proteins were analyzed by western blotting.

To verify the observation from the Connectivity Map analysis that doxycycline inhibits NF-κB target gene expression, we examined the effect of doxycycline treatment on NF-κB activation in DLBCL cell lines. While short (less than 30 minutes) treatment with doxycycline had no inhibitory effect on NF-κB activation in OCI-Ly10 cells (data not shown), an ABC-DLBCL cell line that displays constitutive NF-κB signaling [11, 13], incubation of these cells with doxycycline for 12 hours decreased mRNA levels of several NF-κB targets (Figure 1A), which had been shown previously to be regulated by NF-κ B in these cells (cyclin D2, EBI3 and IκBα) [13, 14], or exhibited the greatest response to doxycycline treatment among the queried NF-κB targets in the cMAP database (MCL-1). The decreases in these mRNAs likely resulted from an inhibition of NF-κB signaling, rather than the consequence of cell death, as the cell viability was not affected by doxycycline at this time point (Figure 1B). Doxycycline treatment also reduced NF-κB reporter activity in OCI-Ly10 cells (Figure 1C) and the levels of several proteins, known to be regulated by NF-κB (Figure 1D). Moreover, doxycycline treatment of ABC-DLBCL cells resulted in a reduction in IKK phosphorylation and nuclear levels of the NF-κB subunits p65 and c-Rel (Figure 1E and 1F), characteristics of inhibition of NF-κB signaling [28, 29]. In addition to inhibiting constitutive NF-κB signaling, doxycycline inhibited signal-induced NF-κB activation in GCB-DLBCL cell lines (Figure 1G), which exhibit minimum constitutive NF-κB activity [11, 13]. Together, these results confirm our observation from the Connectivity Map analysis that doxycycline inhibits NF-κB signaling.

### Doxycycline inhibits the proliferation and survival of DLBCL cells *in vitro*

**Figure 2:**
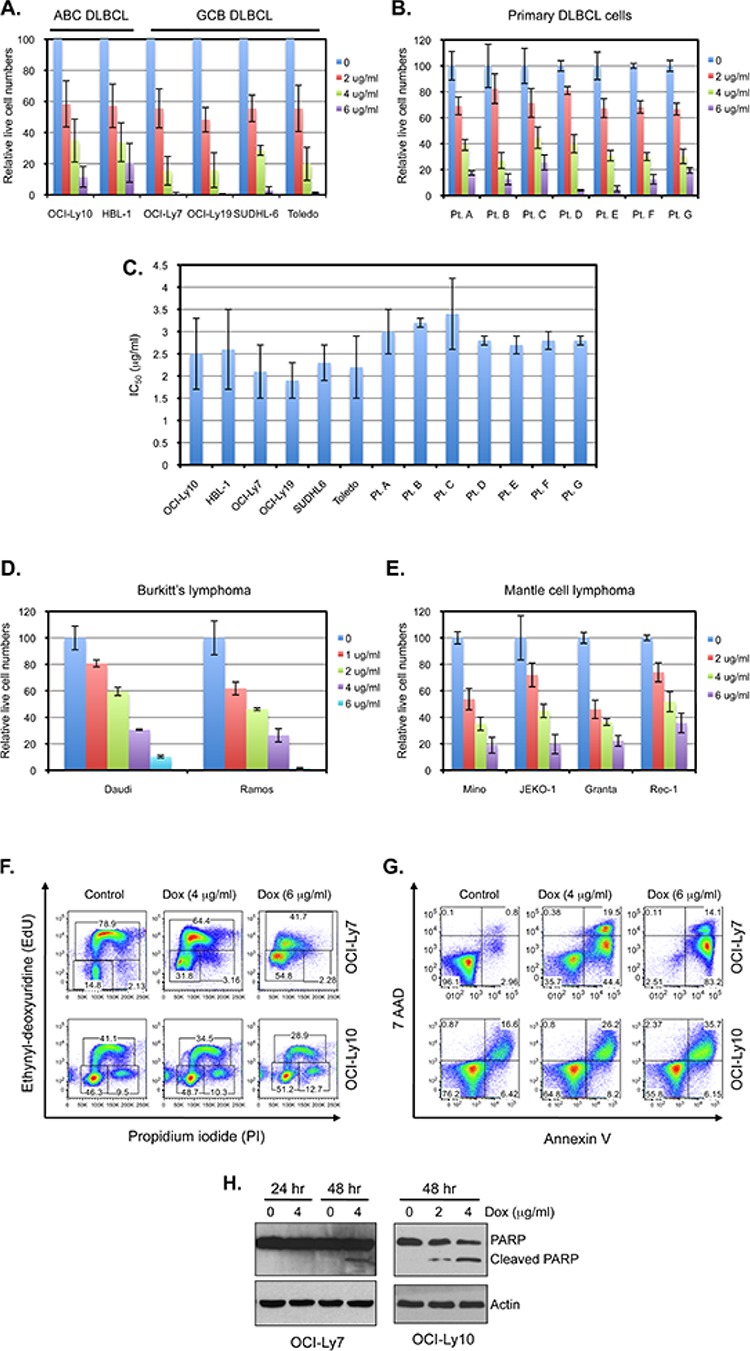
Doxycycline inhibits the proliferation and survival of DLBCL cells **A.** The DLBCL cell lines were treated with the indicated concentrations of doxycycline for 96 hrs. The viable cells were counted by the trypan blue exclusion assay. Shown are the mean and SD from at least three independent experiments. The mean from the samples without exposure to doxycycline was set at 100. **B.** Primary tumor cells from DLBCL patients were plated at 5 × 10^5^ cells/ml for patient samples A–C or at 3 × 10^5^ cell/ml for patient samples D–G and treated with the indicated concentrations of doxycycline for 96 hrs. The live cells were measured as described in (A). The cells from patients A–C were subjected to doxycycline treatment without prior passage *in vitro*, while the cells from patients D-G had been cultured *in vitro* for 3–5 doublings before being treated with doxycycline. Samples D–F and G were classified as GCB and non-GCB subtypes, respectively, by Hans staining. The subtypes for samples A-C were unknown. Mean and SD from triplicate samples are depicted. **C.** The estimated IC_50_ values of doxycycline against DLBCL cell lines and primary cells. The IC_50_ values were calculated from the dose response at 96 hours in experiments described in 2A and 2B. **D.** The Burkitt lymphoma cell lines and **E.** the mantle cell lymphoma cell lines were treated as described in (A). Results from triplicate samples are depicted. **F.** Doxycycline inhibits cell cycle progression. OCI-Ly7 (top panels) and OCI-Ly10 (bottom panels) cells were treated with the indicated concentrations of doxycycline for 48 hrs. Ethynyl-deoxyuridine (EdU) was added into the culture medium for 2 hr before the cells were harvested for cell-cycle distribution analysis. **G.** Doxycycline induces apoptosis of DLBCL cells. OCI-Ly7 (top panels) and OCI-Ly10 cells (bottom panels) were treated with the indicated concentrations of doxycycline for 66 hrs. The apoptotic (annexin V-positive) cells were measured by flow cytometry. **H.** DLBCL cells were treated with doxycycline for the indicated time. The cleavage of PARP1 was analyzed by western blotting.

Since ABC-DLBCL cells depend on constitutive NF-κB signaling for proliferation and survival [11, 13–15], our observation that doxycycline inhibits NF-κB signaling predicts the drug would inhibit the growth of these cells. Indeed, doxycycline treatment for over 24 hours inhibited the growth of ABC-DLBCL cell lines (Figure 2A and 2C, and data not shown). Notably, doxycycline also inhibited the growth of multiple GCB-DLBCL cell lines (Figure 2A and 2C), which do not rely on NF-κB signaling for growth *in vitro* [11, 13–15], suggesting that doxycycline affects other pathways in addition to NF-κB signaling.

As primary DLBCL cells may have different requirements for growth than established cell lines, we examined the effect of doxycycline on the survival of primary DLBCL samples. The viability of primary DLBCL cells was also inhibited by doxycycline, indicating that the cytotoxic effect of doxycycline is not limited to the established cell lines (Figure 2B and 2C).

We also examined the effects of doxycycline on the growth of other types of B-lymphoma cells. We found that the growth of Burkitt lymphoma (Daudi and Ramos) and mantle cell lymphoma (Granta, JEKO-1, Mino and Rec-1) cells were also inhibited by doxycycline at similar concentrations observed for DLBCL cells (Figure 2D and 2E), suggesting that doxycycline inhibits the growth of a broad range of aggressive B-lymphoma cells in culture.

The average peak concentration of doxycycline in human serum is 3–6 μg/ml with a single dose of 200 mg/day, and the peak concentration can be higher with multiple dosing [30–33]. As the elimination half-life of doxycycline in human serum is about 20 hours [34, 35], our results thus suggest that growth of the lymphoma cells *in vitro* is inhibited by a level of doxycycline that is maintained in the sera of human patients receiving a normal dose of the drug.

To investigate the effects of doxycycline on cell proliferation and/or survival, we examined cell cycle distribution and apoptosis of DLBCL cells following drug exposure. Doxycycline treatment resulted in a reduction of DLBCL cells in S phase and an accumulation of cells in G_1_ phase (Figure 2F), indicating that doxycycline inhibits cell cycle progression of DLBCL cells. Doxycycline also increased apoptosis of DLBCL cells, as judged by a doxycycline-induced increase in annexin V-positive cells and an elevated cleavage of PARP (Figure 2G and 2H), hallmarks of apoptosis [36–38]. Therefore, doxycycline inhibits both proliferation and survival of DLBCL cells.

### Doxycycline inhibits tumor growth of ABC and GCB DLBCL cells

**Figure 3:**
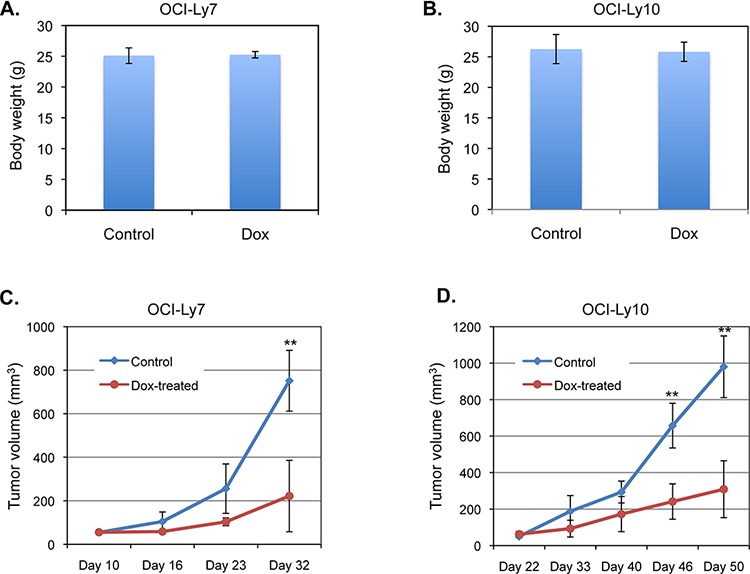
Doxycycline inhibits the growth of DLBCL tumors xenografted in mice Mice bearing tumors from OCI-Ly7 cells **A.** or OCI-Ly10 cells **B.** were injected once daily with either saline (Control) or doxycycline (Dox), starting at day 10 and day 22 after implantation of OCI-Ly7 and OCI-Ly10 cells, respectively. The whole body weight of the mice at completion of the experiments (day 32 and day 50 for OCI-Ly7 and OCI-Ly10, respectively) was measured. The mean and SD are depicted. **C.** The mean and SD of tumor volume from the mice described in (A) are shown. **indicates significant difference between the control group and doxycycline-treated group (*p* < 0.01, *n* = 4 for each group). **D.** The mean and SD of tumor volume from the mice described in (B) are presented. **p < 0.01 (*n* = 4 for each group).

To investigate whether doxycycline inhibits DLBCL tumor growth *in vivo*, we examined the effect of doxycycline on the tumor growth of OCI-Ly7 and OCI-Ly10 cells implanted into immunodeficient mice. The tumor-bearing mice were injected intraperitoneally either with saline as a control or with doxycycline at a dose that was suggested to result in a serum concentration of doxycycline similar to that found in the sera of human patients given the standard dose of the drug [25, 39]. While the administration of doxycycline had no effect on the body weight of the treated mice (Figure 3A and 3B), doxycycline treatment significantly inhibited DLBCL tumor growth (Figure 3C and 3D). We measured the concentrations of doxycycline in the mouse sera 3 hours after the last drug administration and found that the serum doxycycline concentrations varied from 2.3 μg/ml to 5.5 μg/ml in the drug-treated mice (Supplementary Figure 1). These results suggest that doxycycline inhibits DLBCL tumor growth xenografted in mice at concentrations that may be potentially achievable in patient sera with a clinically relevant drug dose.

### Doxycycline inhibits multiple signaling events in DLBCL cells

**Figure 4:**
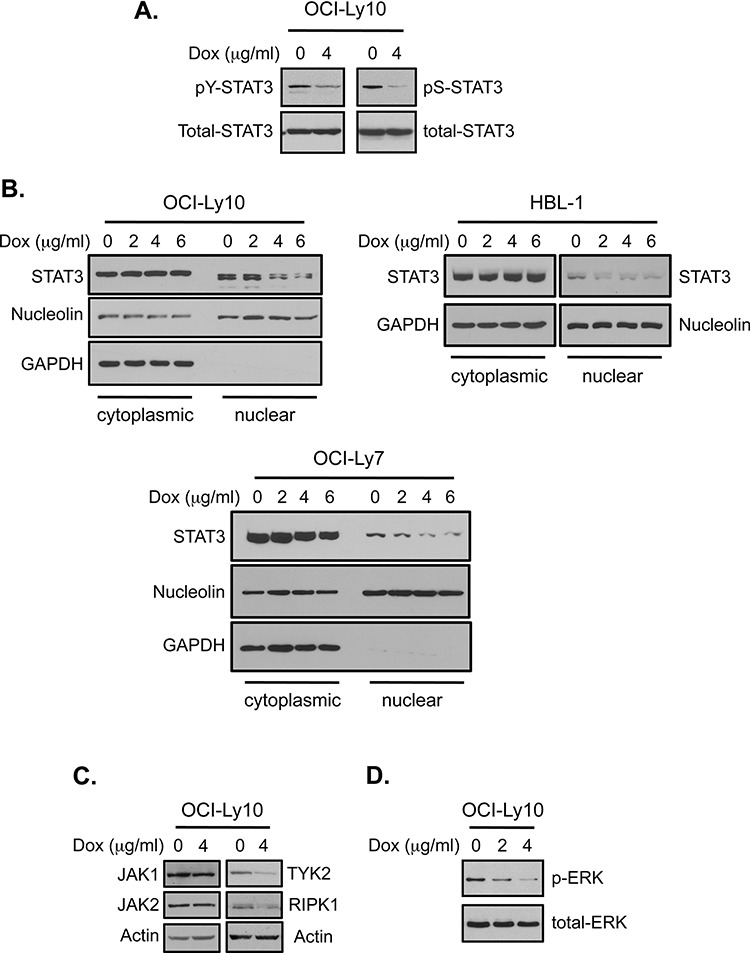
Doxycycline inhibits multiple signaling events in DLBCL cells **A.** OCI-Ly10 cells were treated with the indicated concentrations of doxycycline for 8 hrs. The levels of STAT3 phosphorylation at tyrosine-705 (pY-STAT3) and serine-727 (pS-STAT3) residues were analyzed by western blotting. **B.** Western blot analysis of the STAT3 levels in cytoplasmic and nuclear fractions of the indicated DLBCL cells treated with doxycycline for 24 hrs. The clean nuclear preparations are indicated by the lack of GAPDH signal in the nuclear fractions. The same cytoplasmic and nuclear preparations from HBL-1 cells, described in Figure 1F, were used in this study. **C** and **D.** OCI-Ly10 cells were treated with doxycycline for 4 hrs (C) or 24 hrs (D). The levels of the indicated proteins were analyzed by western blotting. For detection of phospho-ERK, the cells were treated with IL-6 (10 ng/ml) for 15 minutes before the analysis.

It is unlikely that the growth inhibitory effect of doxycycline on DLBCL cells results merely from the inhibition of MMPs, as Prinomastat, a highly potent MMP inhibitor with K_i_ values in the pM to nM range [40], only exhibited inhibitory effects on the growth of DLBCL cell at concentrations about 10, 000 times above the Ki values of the compound (Supplementary Figure 2A). Moreover, expression of several proteins such as MCL-1 and MMP9, shown to be inhibited by doxycycline in DLBCL cells, was unaffected by high concentrations of Prinomastat in these cells (Supplementary Figure 2B). Therefore, we set out to identify the target(s) of doxycycline in DLBCL cells that might be relevant to the observed growth-inhibitory effect of the drug. Since the expression of several NF-κB regulated targets such as MCL-1 and MYC is also regulated by the signal transducer and transcription factor 3 (STAT3) [41], and since there is potent cooperation between NF-κB and STAT3 signaling [42, 43], we investigated the possibility that doxycycline may inhibit STAT3 signaling. STAT3 activity is critically regulated by two phosphorylation events. Phosphorylation of tyrosine-705 is required for STAT3 dimerization and subsequent nuclear translocation, while phosphorylation of serine-727 positively regulates STAT3 transcriptional activity [44, 45]. Interestingly, phosphorylation of both tyrosine-705 and serine-727 of STAT3 was inhibited by doxycycline in OCI-Ly10 cells (Figure 4A). Additionally, doxycycline treatment reduced the nuclear levels of STAT3 protein in DLBCL cells (Figure 4B). Thus, doxycycline also inhibits STAT3 activation in DLBCL cells, apparently through more than one mechanism.

Phosphorylation of tyrosine-705 of STAT3 is carried out by members of the Janus kinase (JAK) family, which includes JAK1, JAK2, JAK3 and TYK2 [45]. Treatment of OCI-Ly10 cells with doxycycline for a short time (4 hours) decreased the protein levels of JAK1, JAK2 and TYK2 at varying degrees (Figure 4C). STAT3 serine-727 phosphorylation can be mediated by different kinases, including RIP1K and ERK, depending on cellular context [46, 47]. As shown in Figure 4C and 4D, doxycycline treatment inhibited both RIP1 expression and ERK activation in DLBCL cells. Collectively, our results suggest that doxycycline exhibits pleotropic effects in DLBCL cells.

### Doxycycline appears to affect HSP90 chaperone function in DLBCL cells via an indirect mechanism

**Figure 5:**
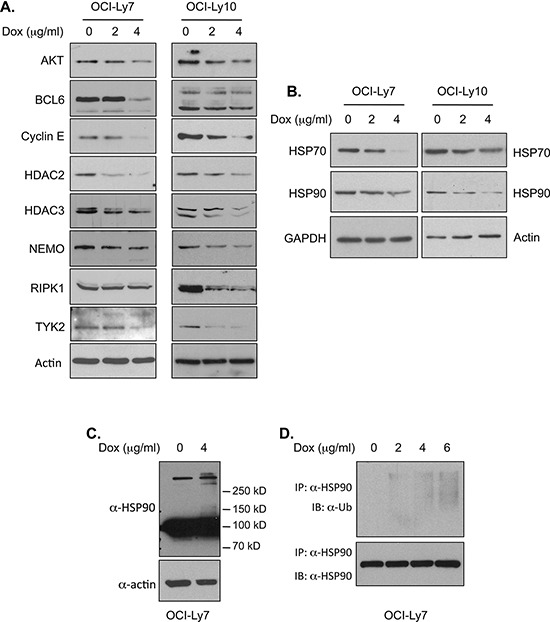
Doxycycline treatment reduces HSP90 activity in DLBCL cells **A.** Doxycycline treatment decreases the levels of HSP90 client proteins in DLBCL cells. The DLBCL cells were treated with the indicated concentrations of doxycycline for 24 hrs. The levels of HSP90 client proteins in these cells were analyzed by western blotting. **B.** Reduced protein levels of HSP70 and HSP90 in DLBCL cells treated with doxycycline. DLBCL cells were treated with doxycycline for 24 hrs, and the levels of HSP70 and HSP90 were analyzed by western blotting. **C.** Doxycycline treatment leads to HSP90 protein modification. Western blotting analysis of HSP90 protein in OCI-Ly7 cells treated with doxycycline for 2 hrs. **D.** Doxycycline induces HSP90 ubiquitination. The cell lysates from OCI-Ly7 cells were subjected to immunoprecipitation with an HSP90-specific antibody. The products were then analyzed by western blotting with a ubiquitin antibody (top panel) or an HSP90 antibody (bottom panel).

The molecular chaperone heat shock protein 90 (HSP90) promotes the folding and function of a large number of substrate proteins, referred to as HSP90 clients, many of which are crucial regulators of diverse cellular functions [48–50]. Dysregulation of HSP90 expression and activity has been observed in a variety of cancers including DLBCL [51, 52]. HSP90 inhibitors can induce proliferation arrest and apoptosis in DLBCL cells [53, 54]. Inhibition of HSP90 activity in cancer cells results in degradation of client proteins, such as TYK2 and RIPK1, and reduction in NF-κB signaling, STAT3 phosphorylation and ERK activation [55–60], the changes seen in DLBCL cells treated with doxycycline (Figures 1 and 4). We therefore investigated the possibility that doxycycline might affect HSP90 chaperone function. Similar to the effects of HSP90 inhibitors, doxycycline treatment decreased the levels of several known HSP90 client proteins in DLBCL cells (Figure 5A, Supplementary Figure 3A and 4A), while such treatment had no inhibitory effect on the mRNA levels of these proteins (Supplementary Figure 5A). Doxycycline treatment also resulted in reduction of HSP90 client proteins in other types of NHL cells (Supplementary Figure 3A). Moreover, as observed with the HSP90 inhibitor 17-AAG, doxycycline treatment led to a decrease of the RIPK1 protein in OCI-Ly10 cells, but not in OCI-Ly7 cells (Figure 5A and Supplementary Figure 4A). Thus, doxycycline treatment exhibits similar pleotropic and cell-type specific effects as HSP90 inhibitors [61]. Taken together, our results suggest that doxycycline affects HSP90 function.

One class of HSP90 inhibitors, which have been most extensively studied, interact with the nucleotide pocket at the N-terminus of HSP90 and interfere with the conformational changes required for normal HSP90 chaperone function [48]. To test whether doxycycline acts on HSP90 in a similar fashion as these HSP90 inhibitors, we examined whether doxycycline affects the nucleotide pocket of HSP90, using HSP90 binding to GA-beads as the readout [62]. We found that doxycycline has no inhibitory effect on binding of HSP90 to the GA-beads (Supplementary Figure 4C), indicating that doxycycline may affect HSP90 function through a mechanism other than a direct binding to the HSP90 nucleotide pocket. In contrast to the nucleotide pocket-binding HSP90 inhibitors, which increase expression of HSP70 ([48, 63], and Supplementary Figure 4B), doxycycline treatment resulted in a decrease in the levels of HSP70 protein (Figure 5B and Supplementary Figure 3A), further suggesting that doxycycline acts differently than the N-terminal HSP90 inhibitors.

Notably, doxycycline treatment also caused a reduction in the levels of HSP90 protein (Figure 5B and Supplementary Figure 3A), indicating that doxycycline acts differently than common HSP90 inhibitors, which in general have no effect on the levels of HSP90 protein. Prior to the decrease in HSP90 protein, an increase in ubiquitination of HSP90 was observed following doxycycline treatment (Figure 5C and 5D), suggesting that the reduction of this protein may result from proteasome degradation. The modification of HSP90 as well as the reduction in the levels of HSP70 and HSP90 protein may contribute to the decreased HSP90 function in DLBCL cells. Thus, doxycycline appears to interfere with HSP90 function through an indirect mechanism. Similarly, reduction in the levels of HSP70, HSP90 and HSP90 client proteins were also observed in the xenografted DLBCL tumors treated with doxycycline (Supplementary Figure 3B), suggesting that doxycycline may inhibit DLBCL cell growth through similar mechanism(s) *in vitro* and *in vivo*.

### Doxycycline inhibits CSN5 activity *in vitro*

**Figure 6:**
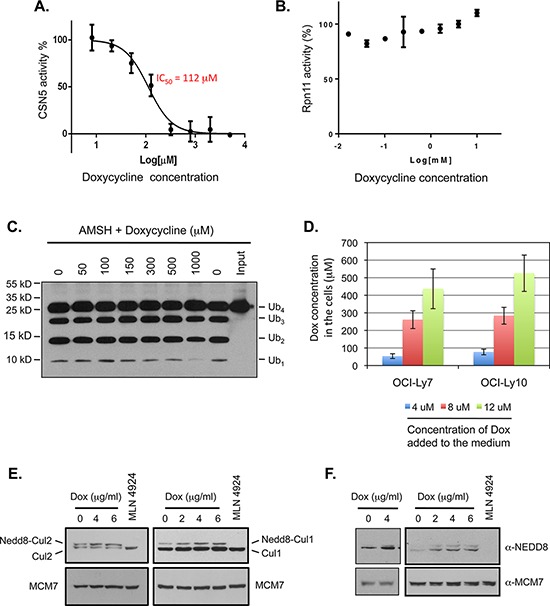
Doxycycline inhibits the deneddylase activity of CSN5 *in vitro* and in DLBCL cells **A.** Doxycycline inhibits CSN5 deneddylase activity *in vitro*. SCF^Skp2^ conjugated with Oregon Green-labeled Nedd8 (5 nM) was incubated with purified CSN complex (0.14 nM) at 30°C in the presence of different concentrations of doxycycline (5000 μM, 2000 μM, 800 μM, 320 μM, 128 μM, 51.2 μM, 20.48 μM, and 8.192 μM). Deconjugation of Nedd8 was monitored by a decrease in fluorescence polarization using a Pherastar fluorescence plate reader over 40 minutes. Initial reaction rates at different concentrations of doxycycline were estimated from the progress curves to determine the percent of activity remaining, which is plotted against doxycycline concentration. **B.** Doxycycline has no inhibitory effect on Rpn11 activity *in vitro*. 5 nM model substrate Ub4-pepOG was incubated with 3 nM 26S proteasome in the presence of the indicated concentrations of doxycycline at 30°C. The polarization was monitored using a Pherastar fluorescence plate reader for 40 minutes. The rate of deubiquitination (Rpn11 activity) is determined by linear regression of the polarization value and normalize to the DMSO control. **C.** The effect of doxycycline on AMSH activity *in vitro*. Purified AMSH was incubated with K63-linked tetra-ubiquitin (Ub_4_) in the presence of the indicated concentrations of doxycycline. The reaction products were analyzed by western blotting using a ubiquitin-specific antibody. **D.** Accumulation of doxycycline in DLBCL cells. OCI-Ly7 and OCI-Ly10 cells were cultured in the presence of the indicated concentrations of doxycycline for 4 hrs. The doxycycline amount in the cells was determined by mass spectrometry. The intracellular doxycycline concentrations were calculated based on the cell diameters of 17.1 μm and 13.9 μm for OCI-Ly7 and OCI-Ly10 cell, respectively. **E.** Effect of doxycycline treatment on neddylation of cullin proteins in DLBCL cells. OCI-Ly7 (left panels) and OCI-Ly10 (right panels) cells were treated with the indicated concentrations of doxycycline for 30 minutes. The cell lysates were immunoblotted with antibodies specific for Cullin-1 and Cullin-2, respectively. Analysis of cells treated with MLN4924, a specific inhibitor of the Nedd8-activating enzyme (NAE), was included to confirm the identities of the slow-migrating forms of the Cullin proteins. **F.** Doxycycline treatment increases protein neddylation in DLBCL cells. DLBCL cells were treated as described in (E). The cell lysates were subject to western blot analysis with an anti-NEDD8 antibody. Analysis of OCI-Ly10 cells treated with MLN4924 was included as an antibody specificity control.

In addition to increased ubiquitination of HSP90, we frequently detected small increases in total cellular protein ubiquitination in DLBCL cells treated with doxycycline (Supplementary Figure 5B). These observations led us to consider the possibility that doxycycline may target a molecule that regulates the levels of protein ubiquitination. Interestingly, members of the zinc-dependent JAMM family of metalloproteinases are deubiquitinating or deneddylating enzymes that play important roles in various cellular processes [64–68]. Given that doxycycline is a known zinc chelator and inhibits MMPs through chelating the essential zinc ions of these enzymes [23], it is possible that doxycycline may inhibit JAMM family metalloproteinases through a similar mechanism. Among the six characterized members of the JAMM family, CSN5 functions as a deneddylating enzyme in the COP-9 signalosome (CSN) complex [69], while the other members of the family are deubiquitinating enzymes that preferentially cleave lysine 63-linked polyubiquitin chains. Because the CSN complex regulates the activities of a large number of cullin-RING E3 ubiquitin ligases (CRLs) through deneddylation of cullin proteins, a change of CSN5 activity will likely affect protein ubiquitination in the cells [70, 71]. Therefore, we examined the effect of doxycycline on the activity of CSN5. We developed an *in vitro* assay in which deneddylation of Cullin-1 in the SCF^Skp2^ complex by the purified CSN complex is assessed. In this *in vitro* system, doxycycline inhibited CSN5 catalyzed-deneddylation with an IC_50_ about 110 μM (Figure 6A).

To investigate the specificity of doxycycline inhibition among the members of the JAMM family, we examined the effects of doxycycline on the activities of Rpn11 (Poh1) and ASMH *in vitro*. In contrast to the inhibition of CSN5, doxycycline had no inhibitory effect on the activity of Rpn11 up to 10 mM concentration (Figure 6B), nor did it inhibit the activity of AMSH at a concentration up to 500 μM (Figure 6C). The inhibition of AMSH activity seen at 1mM concentration likely resulted from precipitation caused by the high level of doxycycline in the reaction mixture, rather than from a specific enzymatic inhibition. Thus, doxycycline selectively inhibits CSN5, instead of being a general inhibitor of the JAMM family, likely through a mechanism more than mere zinc chelation.

### Doxycycline accumulates in DLBCL cells and inhibits CSN5 function in these cells

We observed significant growth inhibition of DLBCL cells at doxycycline concentrations below 6 μg/ml (approximately 12 μM) (Figure 2). The fact that doxycycline inhibited CSN5 activity significantly *in vitro* only at relatively high drug concentrations (IC_50_: ~110 μM) thus raised the question whether the concentrations of doxycycline in the DLBCL cells under our experimental conditions were sufficient to inhibit CSN5 activity. To address the issue, we measured the doxycycline concentration in DLBCL cells treated with the drug. Notably, doxycycline was enriched in DLBCL cells up to more than 40 fold, compared to the drug concentrations added initially to the medium (Figure 6D). Therefore, the cellular concentrations of doxycycline under the experimental conditions reached a level sufficient to inhibit CSN5 activity in the DLBCL cells.

To investigate whether doxycycline indeed inhibits the activity of CSN5 in DLBCL cells, we examined the effect of doxycycline treatment on cullin neddylation. We previously showed that neddylation of cullin proteins causes a mobility shift that can be detected on western blots and that loss of CSN5 function leads to an increase in neddylated cullin proteins [72, 73]. We thus used this assay to assess the effect of doxycycline on CSN5 activity. Doxycycline treatment resulted in an increase in the neddylated (slower-migrating) forms of Cullin-1 and Cullin-2 in the DLBCL cells (Figure 6E and Supplementary Figure 6), indicating that doxycycline inhibits CSN5 activity in DLBCL cells.

We also examined the effect of doxycycline on neddylation using a NEDD8-specific antibody, which recognizes neddylated proteins in the 80–95 kDa range [74]. Doxycycline treatment increased protein neddylation in DLBCL cells (Figure 6F and Supplementary Figure 6). Collectively, the results support the idea that doxycycline inhibits CSN5 function in cultured DLBCL cells.

### Knockdown of CSN5 impairs the survival of DLBCL cells

**Figure 7:**
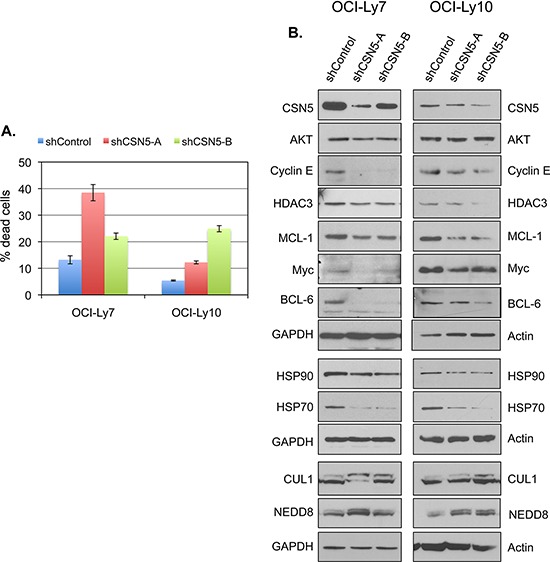
CSN5 is required for the survival in DLBCL cells **A.** DLBCL cells were infected with lentiviruses that express shControl, shCSN5-A or shCSN5-B. Seventy-two (for OCI-Ly7) or 96 hours (for OCI-Ly10) after infection, the viability of the infected cells, which express GFP from the viral vector, were analyzed by flow cytometry. Shown are mean and SD from a representative experiment with triplicate samples. **B.** DLBCL cells were infected with the indicated shRNA-expressing lentiviruses. The levels of the indicated proteins in the infected cells were analyzed by western blotting.

The observations presented above suggest that doxycycline may exert its growth inhibitory effect on DLBCL cells through inhibition of CSN5. This view predicts that CSN5 is required for the survival of DLBCL cells. To test this predication, we examined the effects of CSN5 knockdown in DLBCL cells. Depletion of CSN5 led to marked increases in DLBCL cell death (Figure 7A), demonstrating that CSN5 is an essential survival factor for DLBCL cells. As observed with doxycycline treatment, CSN5 knockdown resulted in reduction in the levels of several HSP90 client proteins as well as HSP70 and HSP90 proteins in both DLBCL cells (Figure 7B) and non-lymphoma cells (Supplementary Figure 7). Thus, CSN5 depletion exhibits the biological and biochemical effects shown for doxycycline treatment in DLBCL cells, consistent with the suggestion that CSN5 is a critical target of the antineoplastic action of doxycycline in DLBCL cells.

## DISCUSSION

This work demonstrates that doxycycline accumulates in DLBCL cells and exhibits potent growth inhibitory activity towards DLBCL cells both *in vitro* and in mouse xenograft models. In addition, we show that doxycycline affects several oncogenic signaling pathways, including the NF-κB, STAT3, ERK and AKT pathways, critical for lymphomagenesis in DLBCL cells. Moreover, the present study identified the deneddylating activity of the COP-9 signalosome CSN5, which is essential for DLBCL cell survival, as a novel target of doxycycline. Together, our results suggest that doxycycline may represent a potential therapeutic agent for DLBCL.

Inhibition of CSN5 deneddylating activity by doxycycline may cause downstream effects through several pathways. Our data suggest that HSP90 levels and function are affected as a result of impairment of CSN5 activity, which may in turn affects downstream oncogenic signaling pathways. As neddylation plays a key role in the regulation of the activities of the CRL E3 ubiquitin ligases, the effects of doxycycline in the lymphoma cells may also result from perturbations in the functions of these enzymes. Additionally, inhibition of CSN5 may affect the function of signaling molecules whose activities are regulated by direct protein neddylation.

Alternatively, given that DLBCL cells accumulates doxycycline to such high concentrations (Figure 6D), it may be possible that doxycycline acts in these cells through additional targets independent of CSN5, although our data are inconsistent with the idea that MMP inhibition is responsible for the growth inhibition by doxycycline in these cells (Supplementary Figure 2). Regardless of whether doxycycline may have additional targets in DLBCL cells, our work presented here indicates that CSN5 is a critical target of doxycycline in DLBCL cells. In addition, our work suggests that CSN5 may represent a potential therapeutic target in DLBCL.

How doxycycline exerts its non-antibiotic activities in human diseases has not been fully elucidated, although in some cases this has been attributed to the ability of doxycycline to inhibit the expression and activities of MMPs and inflammation. In addition, the mechanisms underlying the inhibition of MMP expression and inflammation by doxycycline have not been identified. As NF-κB and STAT3 are major transcriptional regulators of MMPs and inflammatory cytokines, our findings that doxycycline treatment impairs the activation of NF-κB and STAT3 provide new insights into mechanisms of doxycycline action in human diseases.

In a brief communication published previously, it was reported that doxycycline injected intraperitoneally at a dosage of 25 mg/kg/day failed to inhibit the tumor growth of DLBCL xenografted in mice [75]. This study employed six-fold lower doxycycline dosage than what was used in our study. Achieving sufficient *in vivo* concentrations of doxycycline will thus be critical for effective inhibition of lymphoma growth.

Our preclinical studies presented here suggest that doxycycline has antineoplastic activity in DLBCL, as well as in several other types of NHL. Remarkably, doxycycline inhibited growth of all tested DLBCL cells, including cells that are resistant to several currently-tested inhibitors that target HSP90 or the upstream regulators of the BCR signaling pathway in B-cell lymphomas [53, 76]. As doxycycline interferes with the survival pathways in DLBCL cells, it may sensitize the cancer cells to chemotherapy agents. Indeed, we have observed that doxycycline exhibited cooperative cytotoxic effects on DLBCL cells with several chemotherapeutic agents (data not shown). These observations, together with the fact that doxycycline is concentrated in lymphoma cells, raise the possibility that doxycycline may represent a safe and inexpensive drug for NHL therapy either as a single agent or, given the minimal toxicity profile, in combination with standard chemotherapy or rationally targeted agents.

Our results suggest that doxycycline exhibits anti-DLBCL activities *in vitro* and in mouse xenograft models at concentrations that may be achievable in the blood of patients receiving a therapeutic dose of the drug. It should be noted, however, that human cancer in a clinical setting may respond differently than cultured cancer cells or mouse models to the treatment of a drug. Whether doxycycline has therapeutic efficacy in DLBCL patients needs to be tested in clinical studies. Based on our observations, a clinical trail of single agent doxycycline for patients with relapsed or refractory NHL is now ongoing (NCT02086591).

## MATERIALS AND METHODS

### Cells, chemicals, antibodies and buffers

DLBCL cell lines HBL-1, OCI-Ly7, OCI-Ly10, OCI-Ly19, SUDHL-6 and Toledo were cultured as previously described [77]. Primary DLBCL cells were prepared as previously described [77]. All procedures with primary DLBCL cells were carried out with a protocol approved by the University of Rochester Research Subjects Review Board. Burkitt lymphoma cell lines Ramos and Daudi, mantle cell lymphoma cell lines Mino, JEKO-1, Granta and Rec-1 were grown in RPMI medium supplemented with 10% FBS.

Doxycycline was purchased from Sigma, and a stock solution of 10 mg/ml or 40 mg/ml was prepared and stored at −20°C until use. The NAE inhibitor MLN4924 was from Active Biochem. The zinc chelator and CSN5 inhibitor OPT (1, 10-o-phenanthroline) was from Acros Organics. The HSP90 inhibitor 17-AAG was from Selleck Chemicals. The MMP inhibitor Prinomastat was from Sigma.

Antibodies specific for actin (sc-1616), Bcl-6 (sc-858), CUL-1 (sc-11384), Cyclin D2 (sc-452), HDAC2 (sc-7899), HDAC3 (sc-11417), HSP90α/β (sc-7947), IκBα (sc-371), IKKα (sc-7606), CSN5/JAB1 (sc-9074), Mcl-1 (sc-819), MCM7 (sc-22782), MMP9 (sc-6841), NEMO/IKKγ (sc-8032), p65 (sc-372), PARP-1 (sc-7150), c-Rel (sc-70), RIPK1 (sc-7881), STAT3 (rabbit, sc-482), STAT3 (mouse, sc-7179), TGFβR1 (sc-398), TGFβR2 (sc-220), and ubiquitin (sc-8017) were from Santa Cruz Biotechnology. Antibodies specific for AKT (9272), Bcl-xL (2762), CUL-4A (2699), ERK (4695), HSP90β (5087), JAK1 (3344), JAK2 (3230), c-Myc (9402), NEDD8 (polyclonal, 2745), NEDD8 (monoclonal, 2754), nucleolin (12247), phospho-ERK (9101), phospho-IκBα (9241), phospho-IKKα/β (2694), phospho-STAT3 (Tyr705, 9145), phospho-STAT3 (Ser727, 9134), STAT3a (8768), and STAT3 (9139) were from Cell Signaling Technology. CUL-2 antibody was from Invitrogen. GAPDH antibody was from Sigma. Antibodies specific for HSP70 (610607), JAK1 (J24320) and TYK2 (T20220) were from BD Biosciences.

For regular western blotting analysis, cells were lysed in RIPA buffer [78] with proteinase and kinase inhibitors [79]. For the analysis of protein neddylation, the cell lysis buffer contained 2 mM zinc chelator and CSN5 inhibitor OPT (1, 10-o-phenanthroline) [66].

### Connectivity map analysis for drugs that potentially inhibit NF-κB signaling

To indentify compounds that may antagonize NF-κB target gene expression, we queried the Connectivity Map (http://www.broadinstitute.org/cMAP) with the following thirteen NF-κ B target genes [80] as the up-regulated genes: BIRC3, TNFAIP3, NFKB2, IL2RG, NFKB1E, RELB, NFKB1A, CD74, PLEK, MALT1, WNT10A, IRF4, and MCL1. For running the analysis program, two EZH2 target genes [81] CXL12 and CDH6 were included as the down-regulated genes.

### Analysis of mRNA expression of NF-κ B target genes and HSP90 client proteins by quantitative RT-PCR

OCI-Ly10 cells, plated at 3 × 10^5^ cell/ml, were treated with doxycycline for the indicated times. Total RNA from the cells was isolated and treated with DNase I using RNeasy Kit (Qiagen). Standard reverse transcriptase reactions were carried out using reagents from Invitrogen following the manufacturer's suggestion. Two-Step Quantitative PCR reactions with SYBR Green were performed in triplicate on a Bio-Rad MyIQ thermalcycler. For quantitation, expression of each gene was normalized to the level of RhoA. The normalized mRNA levels in the cells not treated with doxycycline were set as 1. The following primer sets were used for the PCR reactions:

**Table:** 

Genes	Primers
Cyclin D2	5′-ATGGTGGTGTCTGCAATGAA-3′ 5′-ATTGAACCATTTGGGATGGA-3′
EBI3	5′-TGTTCTCCATGGCTCCCTAC-3′ 5′-AGCTCCCTGACGCTTGTAAC-3′
IκBα	5′-GCCATTGTAGTTGGTAGCCTTCA-3′ 5′-CTCCGAGACTTTCGAGGAAATAC-3′
MCL-1	5′-AGTCCCGTTTTGTCCTTACGA-3′ 5′-GTGCCTTTGTGGCTAAACACT-3′
RhoA	5′-TGGAAAGACATGCTTGCTCAT-3′ 5′-GCCTCAG GCGATCATAATCTTC-3′
AKT	5′-cgacgtggctattgtgaagg-3′ 5′-ttgaggaggaagtagcgtgg-3′
BCL6	5′-taaaacggtcctcatggcct-3′ 5′-atctctgcttcactggcctt-3′
HDAC2	5′-ATAAAGCCACTGCCGAAGAA-3′ 5′-TCCTCCAGCCCAATTAACAG-3′
HDAC3	5′-ggagctggacaccctatgaa-3′ 5′-gactcttggtgaagccttgc-3′
NEMO	5′-aggtggagcacctgaagaga-3′ 5′-cagagcctggcattccttag-3′
RIPK1	5′-ccgagatgagtactccgctt-3′ 5′-ccattcttcttagcggtgcc-3′
TYK2	5′-gcatttctaccagaggcagc-3′ 5′-ggtcggatcgtagcagtaca-3′

### NF-κB-luciferase reporter OCI-Ly10 cell line

The NF-κB reporter OCI-Ly10 cell line was generously provided by Dr. John Aston (University of Rochester). The cells carry a stably integrated firefly luciferase reporter under the control of 5 copies of the NF-κB response element (Invitrogen). The cells exhibit high basal luciferase activity, responding to both NF-κB activating (such as TNFα and PMA) and inhibiting (IKK inhibitors) factors.

### Analyses of cell viability, apoptosis and cell proliferation

Cells were plated in triplicates at a concentration of 2–3 × 10^5^ cells/ml. Doxycycline (dissolved in sterile water) was added into the culture medium, and the cells were incubated at 37°C. Forty-eight hours after incubation, an equal volume of fresh medium with the original concentrations of doxycycline was added. Cell viability was measured by Trypan blue exclusion assay [77]. Apoptosis was assayed as previously described [82]. Analysis of the cell-cycle distribution was carried out essentially as described previously [83], except that 5-ethynyl-2′-deoxyuridine (EdU) was used in place of bromodeoxyuridine (BrdU). Analysis of EdU incorporation into DNA was performed with a Click-it EdU flow cytometry assay kit (Invitrogen).

### *In vivo* xenograft studies

All animal experiments were performed with protocols approved by our Institutional Animal Care and Use Committee. Seven-week-old female NSG mice (Stock^#^: 005557, The Jackson Laboratory) were subcutaneously injected with OCI-Ly7 cells (5 × 10^6^) and OCI-Ly10 cells (3 × 10^6^), respectively. When tumors were palpable (40–85 mm^3^), the tumor-bearing mice were randomized and injected intraperitoneally once daily with either sterile saline as controls or doxycycline prepared in saline. The animals were given a doxycycline dose of 95 mg/kg/day for the first two days. Then a dose of 150 mg/kg/day was administered afterwards. The tumor size was measured with a digital caliper, and tumor volumes were calculated according to the formula [84]: tumor volume = π/6 × (length) × (width) × (height). Statistic significance of the difference was analyzed by standard student *t* test.

### Measurement of doxycycline concentrations

For measuring doxycycline concentrations in mice sera, mice were sacrificed 3 hours after the last administration of doxycycline. Blood was collected by cardiac puncture and incubated at 4°C for 2 hours. The sera were obtained by centrifugation as previously describe [78] and stored at −80°C until analysis. The serum samples (15 μl) were mixed with 10 volumes of acetonitrile in the Eppendorf LoBind tubes and vortexed for 2 minutes at room temperature. The samples were centrifuged at 18, 000 g for 5 minutes. The supernatants were collected and dried down under a steam of nitrogen or in a Speed Vac. The dried samples were dissolved in 50% methanol, centrifuged (18, 000 g, 2 minutes) to remove any debris if necessary and analyzed by liquid chromatography tandem mass spectrometry (LC-MS/MS) at the University of Rochester Proteomics Center. For calculation of extraction efficiencies, sera from untreated mice were spiked with appropriate amounts of doxycycline and processed as described for the doxycycline-containing serum samples.

For measuring intracellular concentrations of doxycycline, cells (3–3.3 × 10^5^ cells/ml) were incubated with various concentrations of doxycycline for 4 hours. The cells (4.6–5 × 10^6^ cells) were collected by centrifugation at 320 g for 3 minutes. The cell pellets were quickly washed once with 1.4 ml culture medium, collected by centrifugation at 1500g for 2 minute, frozen in a dry ice/ethanol bath and stored at −80°C until analysis. The volume of each cell sample was brought to 40 μl with a solution of 10 mM Tris-HCl, pH 8.0 and 10 mM KCl, and mixed with 10 volumes of acetonitrile. The extraction and mass spectrometry analysis were carried out as described above for the analysis of serum samples. For preparation of the standard curves, cell lysates (40 μl each), prepared from the same numbers of the cells as in the cell samples (4.6–5 × 10^6^ cells) by hypotonic buffer (10 mM Tris-HCl, pH8.0 and 10 mM KCl) treatment and homogenizing in a tight fitting Dounce homogenizer, were spiked with varying amounts of doxycycline. The extraction and mass spectrometry analysis were then carried out as described for cell samples. The data obtained were used to generate the standard curves.

For calculating the intracellular doxycycline concentrations, we determined the volume of the tested cells. The diameters of the cells were measured using a Nikon Eclipse TE300 inverted microscope equipped with an eyepiece grid reticle. The cell volumes were calculated using the formula: V = 4 π/3 × r^3^, where r is the radius of the cells.

### *In vitro* activity assays for CSN5, RPN11 and AMSH

Purification of CSN and generation of Nedd8-conjugated SCF^Skp2^ substrate have been previously described [85]. CSN-mediated deneddylation was measured by monitoring a decrease in fluorescence polarization upon deconjugation of fluorescent Nedd8 from SCF^skp2^ (PubChem Bioassay AID651999). A detailed characterization of the assay is being prepared for publication elsewhere.

The Rpn11 assay is described in PubChem (AID 588493).

The *in vitro* de-ubiquitination activity of AMSH was assayed essentially as previously described [67]. Briefly, GST-AMSH protein was purified from E. coli BL21 cells that harbor the pGEX-AMSH plasmid [67], kindly provided by Dr. Sylvie Urbe, using the method previously described [86]. The purified GST-AMSH was incubated with K63-linked tetra-ubiquitin (Boston Biochem) in the DUB buffer with or without the presence of doxycycline at 37°C for 4 hr. The reactions were terminated with the SDS-PAGE sample buffer, and the products were analyzed by western blotting with an anti-ubiquitin antibody.

### Knockdown of CSN5 expression by RNA interference

The lentiviral pGIPZ constructs expressing shRNAs specific for human CSN5 (shCSN5-A, V3LHS_361326 and shCSN5-B, V3LHS-361327) or a non-silencing shRNA (shControl, RHS4346) were purchased from Thermo Scientific. Production of pseudotyped lentiviruses carrying pGIPZ constructs and infection of DLBCL cells with the viruses were performed as previously described [77, 82].

## SUPPLEMENTARY FIGURES


