# Supplementary material for: Inhibition of COP9-signalosome (CSN) deneddylating activity and tumor growth of diffuse large B-cell lymphomas by doxycycline
Source: Oncotarget. 2015 Jun 4;6(17):14796–813. doi: 10.18632/oncotarget.4193 (PMC4558116; doi:10.18632/oncotarget.4193)
Supplement: Supplementary file 1 [file oncotarget-06-14796-s001.pdf]

SUPPLEMENTARY FIGURES

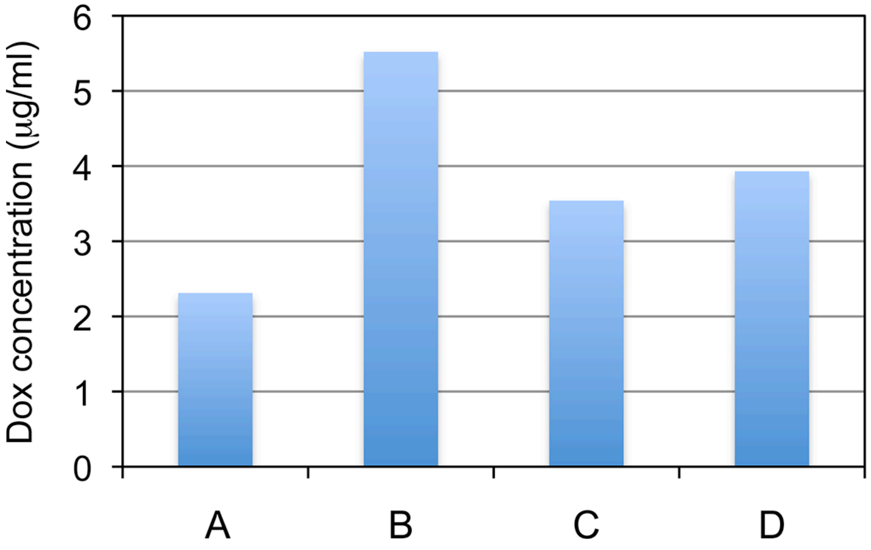

Supplementary Figure 1: Doxycycline concentrations in the sera of the treated mice. Four mice implanted with OCI-Ly7 cells were treated with doxycycline as described in Figure 3. The mice were sacrificed 3 hours after last drug administration, and the serum doxycycline concentrations were measured by mass spectrometry. Shown are the average values from duplicate assays. As the half life of doxycycline was approximately 4 hours, (English (1967) Proc. Soc. Exp. Bio. Med., 126:487-491; Chang et al. (1990) Antimicrob. Agents Chemother. 34:775-780), the serum doxycycline concentrations measured should fall between the peak and trough concentrations.

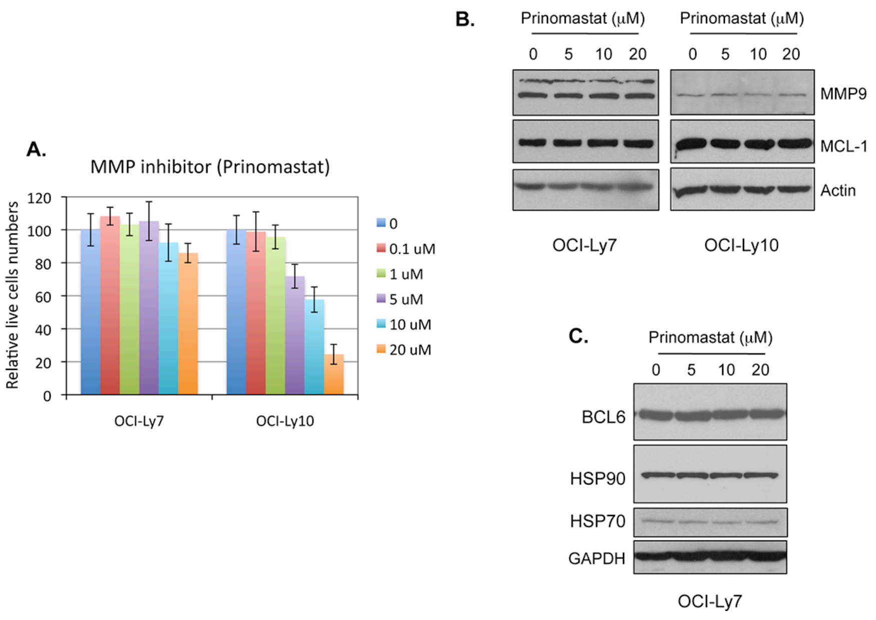

Supplementary Figure 2: The effects of MMP inhibitor Prinomastat on DLBCL cells growth and expression of the selected proteins. A. DLBCL cells were cultured in the presence of the indicated concentrations of Prinomastat. Forty-eight hrs after incubation, an equal volume of fresh medium with the original concentrations of Prinomastat was added. The viability of the cells was analyzed at 96 hrs of incubation by Trypan blue exclusion assay. Shown are the mean and standard deviation (SD) from triplicate samples. **B.** Prinomastat treatment has no inhibitory effect on the expression of MMP9 and MCL-1 proteins. The DLBCL cells were treated with the indicated concentrations of Prinomastat for 24 hrs. The levels of MMP9 and MCL-1 were analyzed by western blotting **C.** Prinomastat does not inhibit the expression of BCL6, HSP70 and HSP90. OCI-Ly7 cells were treated with the indicated concentrations of Prinomastat for 24 hrs. Expression of the indicated proteins was analyzed by western blotting.

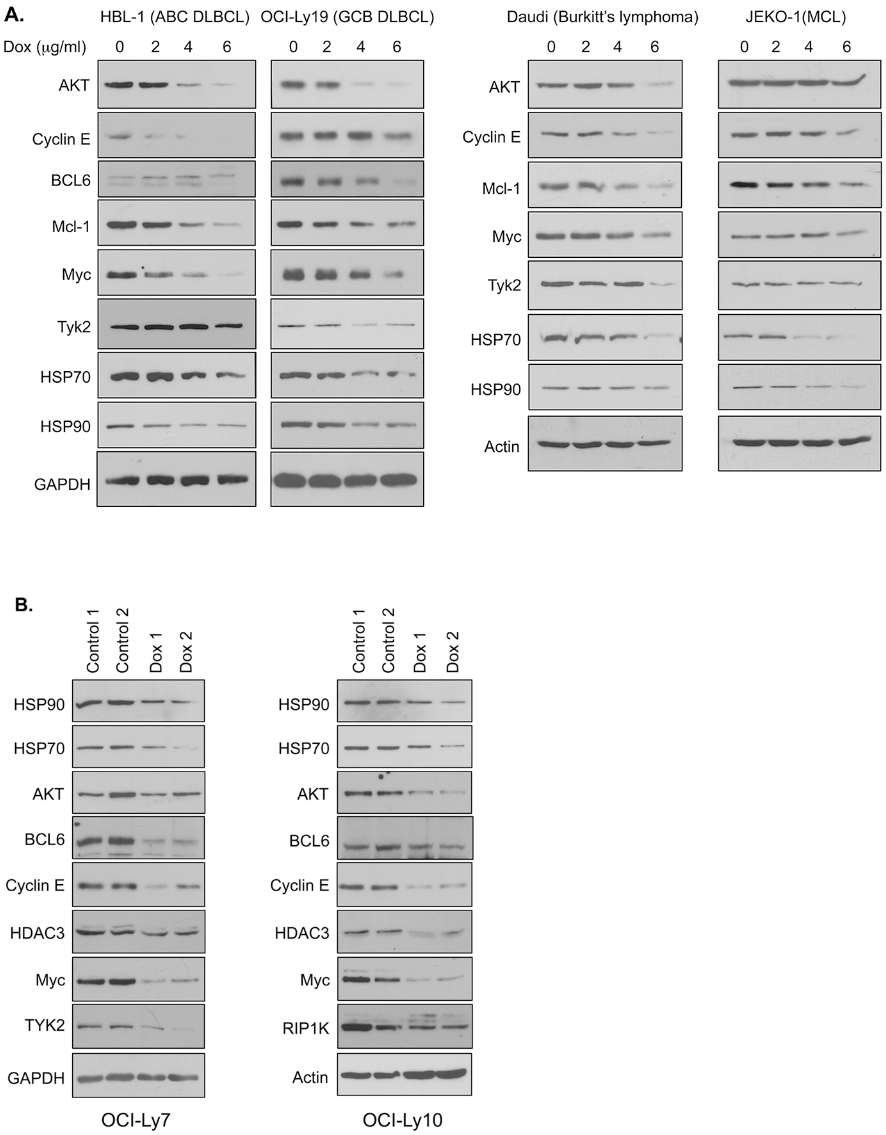

Supplementary Figure 3: Doxycycline affects HSP90 function in multiple NHL cells and in DLBCL tumors xenografted in mice. A. The indicated NHL cell lines were treated as described in Figure 5A. The levels of the indicated proteins were analyzed by western blotting. MCL: mantle cell lymphoma. **B.** Mice bearing tumors from OCI-Ly7 or OCI-Ly10 cells were injected once daily with either saline (Control) or doxycycline (Dox) as described in Figure 3. Tumors were dissected from mice at day 32 and day 50 for OCI-Ly7 and OCI-Ly10 xenografts, respectively. The isolated tumors were minced on ice with scalpels in RIPA buffer and further dissociated by passage through syringe needles. The expression of the indicated protein in the tumors was then analyzed on western blots.

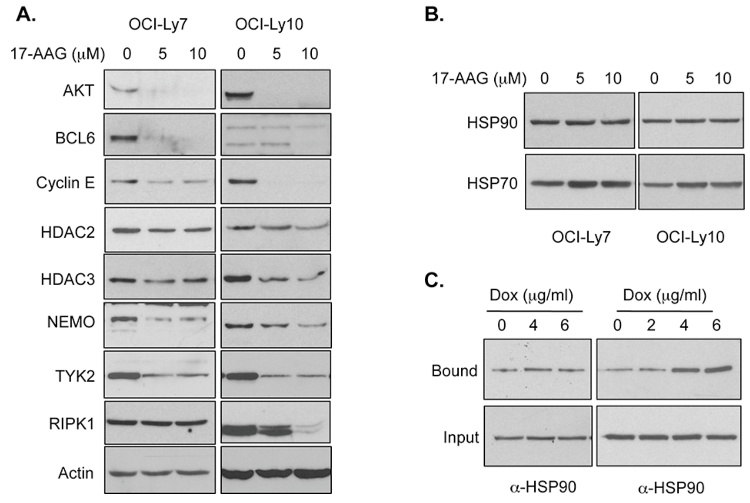

Supplementary Figure 4: Effects of 17-AAG and doxycycline on the expression of HSP90 client proteins, HSP90 and HSP70 in DLBCL cells. A. DLBCL cells were treated with the indicated concentrations of HSP90 inhibitor 17-AAG for 24 hrs. The levels of the HSP90 client proteins were analyzed by western blotting. **B.** DLBCL cells were treated with the indicated concentrations of 17-AAG for 24 hrs. The levels of HSP70 and HSP90 were analyzed by western blotting. Consistent with previous published observations 17-AAG has no significant effect on the levels of HSP90 protein while it increases the levels of HSP70 protein. **C.** Doxycycline does not inhibit binding of HSP90 to the GA-beads. Left panels: Lysates from OCI-Ly7 cells were incubated with biotin-conjugated Geldanamycin (GA) prebound to Straptavidin-Dynabeads (Invitrogen) in the presence of the indicated concentrations of doxycycline at 4°C for 1 hr. The level of HSP90 in the GA-bound fractions was analyzed by western blotting. Right panels: OCI-Ly7 cells were treated with the indicated concentrations of doxycycline for 3 hr. The lysates from the treated cells were incubated with biotin-conjugated GA and Streptavidin-Dynabeads at 4°C for 1 hr. The amount of GA-bound HSP90 was then analyzed by western blotting.

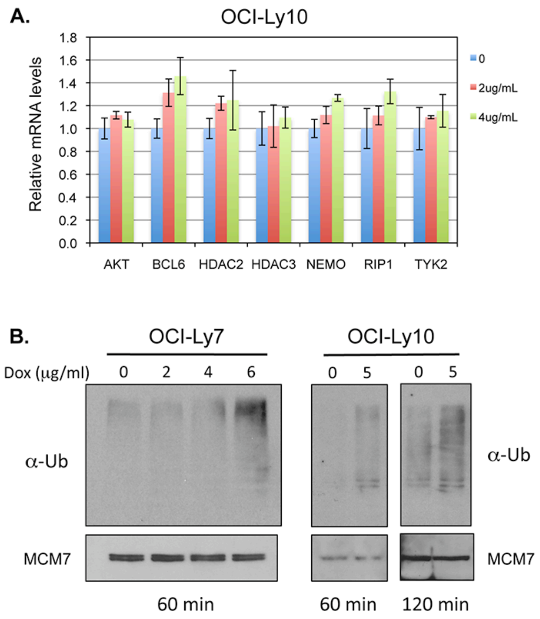

Supplementary Figure 5: Effects of doxycycline treatment on the mRNA levels of HSP90 clients and cellular ubiquitination in DLBCL cells. A. The mRNA levels of the HSP90 client proteins in OCI-Ly10 cells treated with the indicated concentrations of doxycycline for 24 hrs were analyzed by quantitative RT-PCR. The assays were carried out in triplicate. **B.** Doxycycline treatment increases ubiquitination in DLBCL cells. DLBCL cells were treated with the indicated concentrations of doxycycline for 60 min or 120 min. The ubiquitination of cellular proteins was analyzed by western blotting using a ubiquitin-specific antibody.

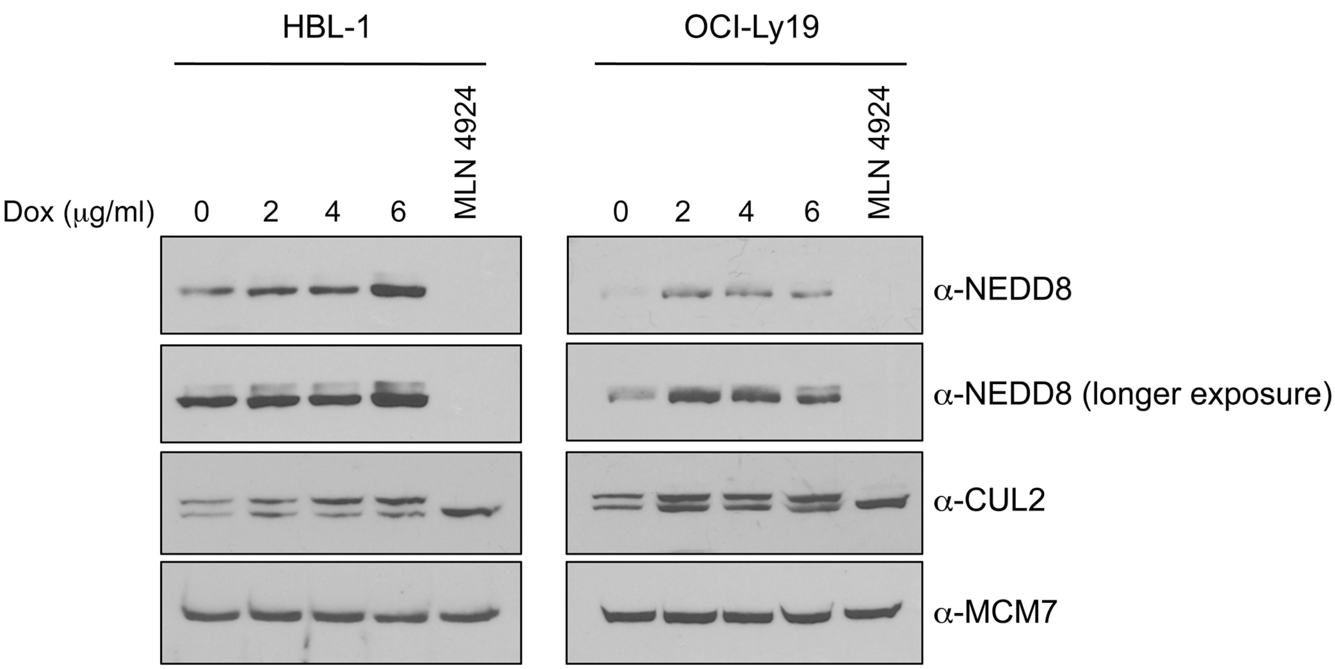

Supplementary Figure 6: Doxycycline treatment increases neddylation in multiple DLBCL cell lines. HBL-1 and OCI-Ly19 cells were treated with the indicated concentrations of doxycycline. Protein neddylation was analyzed as described in Figure 6.

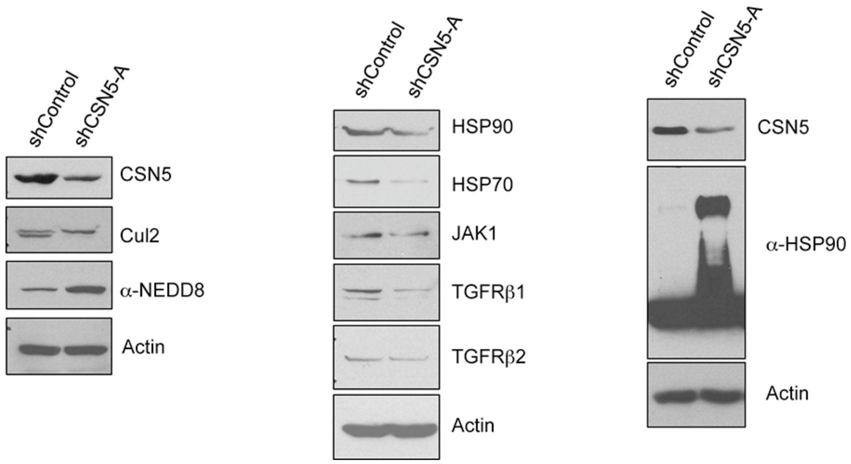

Supplementary Figure 7: CSN5 knockdown in U2OS cells affects protein neddylation and HSP90 function. U2OS cells were infected with lentiviruses that express either shControl or shCSN5-A. The infected cells were selected in the presence of puromycin (2 μg/ml) for 6 days. The levels of the indicated proteins were analyzed by western blotting. JAK1, TGFβR1 and TGFβR2 are known HSP90 client proteins in U2OS cells (Haupt et al (2012) BMC Cancer 12:38). A longer exposure of an HSP90 blot is also shown (right panels).
